# La reconstruction du sourcil par greffon composite du cuir chevelu: une astuce pour faciliter la technique

**DOI:** 10.11604/pamj.2015.21.1.6033

**Published:** 2015-05-01

**Authors:** Mounia El Omari, Samir El Mazouz, Noureddine Gharib, Abdallah EL Abbassi

**Affiliations:** 1 Chirurgie Plastique, Reconstructrice et Esthétique, Hôpital Moulay Youssef, Centre Hospitalier Régional, Rabat, Maroc; 2 Service de Chirurgie Plastique, CHU Ibn Sina, Rabat, Maroc

**Keywords:** Séquelle brulure, reconstruction sourcil, greffe composite, burn sequelae, eyebrow reconstruction, composite graft

## Abstract

Les sourcils jouent un rôle important dans l’équilibre esthétique du visage. Leur reconstruction ou ophriopoïése, après séquelle de brûlure fait partie intégrante du programme de réhabilitation de la face brûlée. Plusieurs techniques ont été décrites. Nous insistons ici sur l'intérêt d'une technique simple, à la portée de tous les chirurgiens, et dont la méthode et les résultats peuvent être améliorés par un dessin bien planifié des zones donneuse et receveuse: la greffe composite prélevée au niveau du cuir chevelu dessinée à l'aide d'un calque du sourcil controlatéral.

## Introduction

Le traitement des séquelles de brûlures du visage constitue un véritable défi pour le chirurgien plasticien. Dans cette quête à la réhabilitation de la face brulée, chaque unité anatomique doit être reconstruite de la manière la plus proche du naturel. L'objectif final est de redonner au patient une apparence acceptable et de lui permettre de retrouver une vie sociale. Pour cela, les principes fondamentaux de la chirurgie plastique sont mis en pratique afin de restaurer au mieux l'architecture de la face brulée. C'est dans ce sens que les méthodes de base telles que, par exemple, les greffes composites de cuir chevelu trouvent tout leur intérêt dans la reconstruction des sourcils. Nous présentons cette technique qui constitue une solution simple, rapide et fiable pour reconstruire un sourcil partiellement ou totalement détruit après une brûlure de la face, en décrivant une méthode simple de dessin permettant de simplifier la technique et d'améliorer les résultats cosmétiques.

## Patient et observation

La technique utilisée a été la greffe composite de cuir chevelu sur mesure à partir d'un calque du sourcil controlatéral.

**Figure 1:**
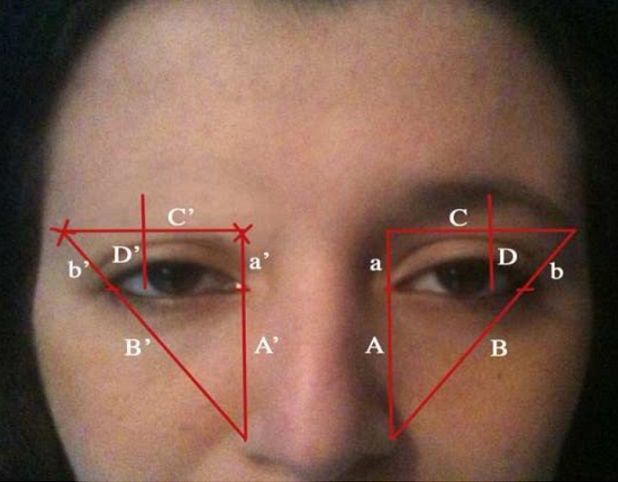
À gauche: repères du sourcil selon Westmore: extrémité médiale du sourcil: sur la ligne verticale entre point le plus latéral du cartilage alaire et canthus interne (A) dont, a= distance canthus interne- extrémité médiale sourcil; extrémité latérale du sourcil: sur la ligne oblique entre point le plus latéral du cartilage alaire et canthus externe (B) dont, b= distance canthus externe- extrémité latérale sourcil. Les 2 extrémités: sur la même ligne horizontale (C); apex: sur la ligne verticale passant par le limbe latéral (D); à droite: Repères de l'emplacement du nouveau sourcil: dessin des lignes (A’), (B’), (C’) et (D’); report des distances a’ = a et b’ = b déterminant ainsi les extrémités du nouveau sourcil

**Figure 2:**
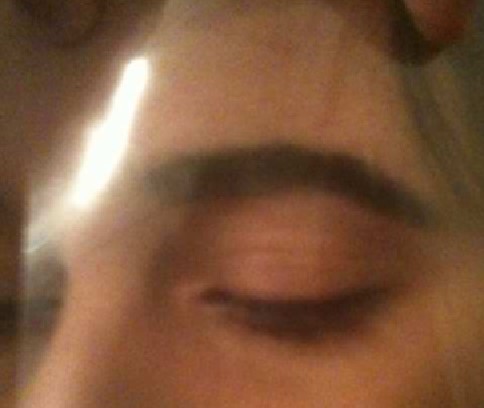
Prise de l'empreinte du sourcil sain après son marquage au crayon gras

**Figure 3:**
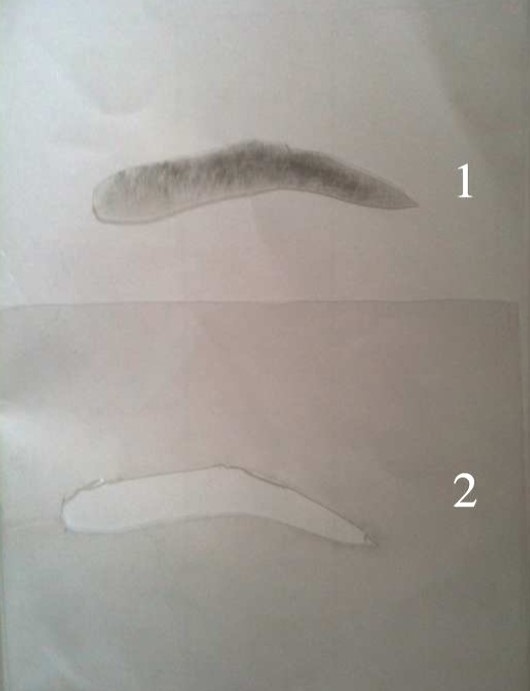
Après découpe: « empreinte » (1) et « pochoir » (2)

**Figure 4:**
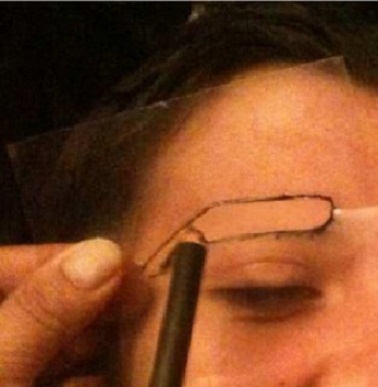
Dessin de la forme du nouveau sourcil à l'aide du « pochoir »

**Dessin:** les dessins du nouveau sourcil (zone receveuse) et de la zone donneuse sont réalisés à partir des repères de Westmore (Figure 1) et d'un patron calqué sur le sourcil controlatéral. Tout d'abord, nous réalisons une empreinte du sourcil sain, en le repassant au crayon gras noir (crayon de maquillage) puis en appliquant dessus un calque (ou autre support transparent, dans le but de pouvoir visualiser cette empreinte même après avoir inversé ce calque) (Figure 2). Si les poils sont trop longs, il est utile de les couper un peu afin d'obtenir uniquement l'empreinte des racines permettant ainsi de déterminer leur direction. Cette empreinte du sourcil est découpée et constitue la référence à partir de laquelle sera choisie la zone donneuse (Figure 3). A partir du négatif obtenu après découpage de l'empreinte, on pourra dessiner la forme du sourcil à reconstruire à la manière d'un pochoir (Figure 4). Quant à son emplacement, il sera déterminé à partir du report des repères du sourcil sain s'il existe en s'aidant des repères de Westmore [1]. Les repères d'un sourcil « idéal » selon les critères de Westmore sont comme suit (Figure 1): l'extrémité médiale se trouve sur la ligne verticale entre le point le plus latéral du cartilage alaire et le canthus interne; l'extrémité latérale se trouve sur la ligne oblique entre le point le plus latéral du cartilage alaire et le canthus externe; les 2 extrémités sont presque sur la même ligne horizontale; l'apex se trouve sur la ligne verticale passant par le limbe latéral.

**Figure 5:**
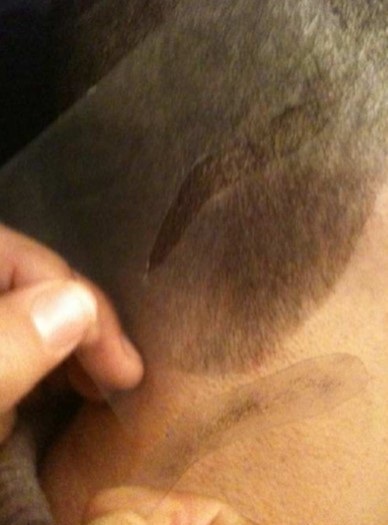
Repérage de la zone donneuse la plus proche de l’ « empreinte » inversée en direction de poils, et dessin à travers le « pochoir »

**Choix de la zone donneuse:** c'est une étape clé dans la technique. De ce choix dépendra en partie le résultat cosmétique final. La bande de cuir chevelu peut être prélevée en retroauriculaire, en temporal ou en occipital. Au préalable, les cheveux seront coupés à ras au niveau de la région choisie. Le choix se fera en se référant à l'empreinte inversée du sourcil sain, afin d'obtenir une bande où les cheveux auront une densité et une orientation proches de celles de l'empreinte. La forme exacte du sourcil ou de la partie du sourcil à prélever sera dessinée au feutre sur la zone donneuse à travers le « pochoir » (négatif de l'empreinte) (Figure 5). Ce dessin sera complété en fuseau à ses extrémités afin d'obtenir une perte de substance suturable.

**Prélèvement:** le prélèvement sera réalisé sous anesthésie locale (xylocaïne simple au niveau de la zone donneuse et receveuse afin d’éviter tout spasme). Les incisions seront bien perpendiculaires au plan cutané pour ne pas couper les follicules pileux obliquement, ce qui les détruirait. Le prélèvement se fera de manière atraumatique. Le greffon composite sera soigneusement dégraissé en préservant les follicules pileux puis mis dans du sérum hépariné avec une expression douce. La zone donneuse sera suturée après hémostase.

**Mise en place du greffon:** tout d'abord on commence par préparer un lit bien vascularisé pour le greffon. Au niveau de la zone receveuse on excise le placard cicatriciel au niveau de la zone dessinée correspondant à l'emplacement du nouveau sourcil et on réalise une hémostase à minima. Le greffon est ensuite mis en place le greffon et suturé aux berges sans tension par des points simples au prolène 5/0.

**Suites opératoires** un pansement gras sera mis en place et ouvert au 5ème jour. L'ablation de fils se fera entre le 5ème et 7ème jour, et le greffon sera régulièrement recouvert par de la vaseline. Le greffon va passer par les différentes phases habituelles de toute greffe composite; blanc, bleu puis rose. Nous n'avons noté dans notre expérience aucune nécrose. Apres une perte partielle transitoire des poils du greffon dans les semaines qui suivent la cicatrisation, on note une repousse ultérieure et une densité définitive à 3mois satisfaisante.

## Discussion

Le sourcil est un élément important de la physionomie du visage. Il joue un rôle fonctionnel dans la protection oculaire (sueur, corps étranger) et surtout un rôle majeur dans l'esthétique et l'expressivité du regard et du visage tout entier. C'est peut-être ce qui explique l'expression ambigüe du visage de la Joconde qui ne semble presque pas avoir de sourcils! Certes, on ne remarque pas les sourcils quand ils existent, mais quand ils n'existent pas, le visage a une apparence inhabituelle. C'est pourquoi la reconstruction du sourcil est une « touche finale » essentielle dans le programme de réhabilitation de la face brulée. Le sourcil est l’élément pileux qui sépare anatomiquement la région du front de la région orbitopalpébrale en croisant le rebord supra-orbitaire. Il existe de nombreuses variations de localisation, de forme, de densité et d'orientation des sourcils selon les individus. Plusieurs auteurs se sont intéressés à définir des critères du sourcil « idéal ». La conception la plus utilisée est celle de Westmore [1]. Ces mesures sont utiles lorsqu'il s'agit de reconstruire les deux sourcils totalement absents. Elles peuvent aider à l’établissement de la forme et de la localisation des nouveaux sourcils, mais il est nécessaire de se référer aux photos antérieures à la brûlure pour adapter le dessin. Par contre, lorsqu'il existe déjà un sourcil sain, il est primordial de prendre celui-ci comme unique référence pour le dessin du sourcil controlatéral manquant, afin d'obtenir le résultat le plus symétrique et donc le plus naturel possible. Le patron obtenu à partir du sourcil controlatéral sera inversé et utilisé comme un « pochoir » pour dessiner l'emplacement du nouveau sourcil. La position exacte sera déterminée en reportant les mesures du coté sain: pour l'extrémité médiale du nouveau sourcil: report de la distance tête du sourcil - canthus interne sur la ligne verticale (Westmore) entre canthus interne et cartilage alaire; pour l'extrémité latérale du nouveau sourcil: report de la distance queue du sourcil - canthus externe sur la ligne oblique (Westmore) entre canthus externe et cartilage alaire;

Les poils du sourcil sont généralement verticaux et orientés légèrement en dehors dans la partie interne, latéralement dans la partie centrale, en dehors et légèrement en bas dans la partie externe. Le respect de l'orientation des poils du sourcil lors de la reconstruction est primordial, afin de lui donner un aspect plus proche du naturel. Pour cela, on peut s'aider de l’ « empreinte » obtenue à partir du sourcil controlatéral pour le choix de la zone donneuse au niveau du scalp retro-auriculaire ou occipital homolatéral qui a une finesse et une orientation capillaire proches de l'empreinte inversée. L'avantage de cet artifice technique « de l'empreinte et du pochoir » est de faciliter le dessin du nouveau sourcil et le choix de la zone donneuse contrairement au dessin subjectif réalisé par certains auteurs en collaboration avec le patient [2]. Tout chirurgien amené à reconstruire un sourcil doit apprécier la complexité de cette région; les poils sont fins, changent de direction selon chaque partie du sourcil, repoussent lentement et la zone de transition entre zone chevelue et zone glabre est discrète. La reconstruction du sourcil ou ophriopoïèse a pour objectif d'obtenir un sourcil comparable au côté sain avec un aspect cosmétique proche du naturel. Plusieurs techniques ont été proposées pour la reconstruction du sourcil après brûlure, le cuir chevelu reste la zone donneuse de choix qu'il s'agisse de greffes ou de lambeaux. La greffe composite à partir d'une bandelette de cuir chevelu est de loin la technique la plus simple, la plus rapide et celle qui autorise le plus de liberté dans le choix de la zone donneuse. Les lambeaux de cuir chevelu à pédicule transitoire ou définitif sont techniquement plus difficiles, nécessitant une dissection soigneuse du pédicule vasculaire, et sont limités dans le choix de la palette chevelue par les exigences vasculaires, et ne semblent pas toujours justifiés au vu des résultats obtenus [3] surtout pour les reconstructions partielles [4]. Leur utilisation peut également être compromise par la mauvaise qualité du cuir chevelu frontal ou temporal habituellement utilisé pour ces lambeaux, qui peut être lui-même brulé [5] ou alopécique (d'origine androgénique) [4]. Dans les reconstructions des deux sourcils, ces techniques sont encore plus lourdes contrairement aux greffes composites [4].

Le lambeau en ilot vascularisé par l'artère temporale superficielle peut nécessiter parfois la réalisation d'un Doppler au préalable [5]. Sa réalisation est techniquement plus difficile et plus longue [6]. Il faut être prudent en s'approchant de la queue du sourcil du fait de la présence de la branche frontale du nerf facial très superficielle à ce niveau. La cicatrice au niveau de la zone donneuse est plus longue [5], et la dissection étendue peut entrainer une alopécie de la zone donneuse [6]. Quand ce lambeau est à pédicule cutané, il doit nécessiter un deuxième temps de sevrage. Lorsqu'il est tunnelisé, la persistance d'une voussure au niveau de la zone de tunnelisation peut être inesthétique [7]. Quant au résultat esthétique final, il est souvent jugé insuffisant voire insatisfaisant [6, 8]. L'anatomie vasculaire conditionne le dessin de la zone de prélèvement du lambeau et laisse donc peu de liberté dans le choix de la palette concernant la direction des cheveux contrairement à la greffe composite [6]. Les variations de l'anatomie vasculaire, notamment l'existence d'un pédicule court peut rendre sa réalisation encore plus complexe pouvant même nécessiter un pontage. L'interruption de l'axe vasculaire temporal est une contre-indication à l'utilisation de ce lambeau [7]. Certains auteurs proposent la reconstruction de sourcil par un lambeau de cuir chevelu frontal ou temporal antérieur à pédicule sous-cutané inferieur [6, 8], qui aurait un résultat cosmétique amélioré. Toutefois, sa réalisation n'est pas dénuée de risques. La dissection doit respecter la branche frontale du nerf facial. Les limites de cette technique sont l'existence d'une brulure associée du front endommageant le fascia sous-cutané ou d'une alopécie androgénique du site donneur [6]. En général, ces lambeaux sont réalisés par des chirurgiens expérimentés [6] sous anesthésie générale [2], alors que la greffe composite est une technique simple à la portée de tout chirurgien débutant [8] et qui peut être réalisée sous simple anesthésie locale [2]. Les sourcils obtenus par ces deux techniques donnent une pilosité trop forte et le sens des poils n'est pas toujours dirigé vers le dehors comme l'esthétique le voudrait donnant un aspect de sourcil « en brosse » [2]. Elles sont plus souvent indiquées pour l'homme qui a des sourcils plus épais. La greffe composite constituerait même, selon certains auteurs, le premier choix pour la reconstruction du sourcil, surtout chez les femmes [5]. Certains procédés peuvent compléter le résultat final obtenu, tels que le tatouage, la lipostructure [3] ou les microgreffes. La dermopigmentation ou tatouage peut être utilisée en complément afin de renforcer l'aspect couvrant en cas de sourcil épais et dense [9]. La lipostructure peut être utile pour redonner à la région sourcilière son galbe naturel. Les microgreffes ont surtout un intérêt dans la correction de petites pertes de substance du sourcil ou en complément d'une autre technique. Leur indication doit rester prudente sur une zone cicatricielle [5]. Utilisées seules pour reconstruire un sourcil complètement détruit après brûlure [2], elles ont certes l'avantage de permettre l'orientation des follicules pileux un par un selon la spécificité de chaque partie du sourcil, mais comparées à la greffe composite d'une bandelette de cuir chevelu, elles demandent plus de précautions, de patience et de temps (environ 100 follicules greffés pour un sourcil [2]) et peuvent nécessiter plusieurs séances avec un résultat esthétique insuffisant. Néanmoins, lorsqu'elles sont utilisées secondairement en complément d'une greffe composite, elles permettent d'améliorer le résultat cosmétique en corrigeant les insuffisances de densité et en affinant la direction de repousse capillaire dans certaines parties du sourcil. Certains auteurs ont même proposé une technique combinant microgreffe et greffe composite simultanément [4]. Nous proposons même d'obtenir ces microgreffons à partir des triangles excisés au niveau de la zone donneuse du cuir chevelu afin d'obtenir un fuseau autofermant. Ils peuvent être transplantés au niveau des berges entre le greffon et la zone receveuse, améliorant ainsi l'aspect de a zone de transition [4].

## Conclusion

La greffe composite est une technique simple, rapide et fiable. Son principal avantage, surtout dans la reconstruction partielle ou totale d'un sourcil, est la liberté de choix d'une zone donneuse proche du sourcil restant en termes de direction des poils et la cicatrice discrète au niveau du site donneur. La reconstruction du sourcil, comme toute reconstruction faciale, requiert une combinaison d'art et de science. C'est pourquoi nous rapportons cette note technique à l'aide d'un « pochoir » et d'une « empreinte » qui peut faciliter le dessin de la zone receveuse et le choix de la zone donneuse pour un meilleur résultat esthétique.
